# The Impact of the Built Environment on Young People’s Physical Activity Patterns: A Suburban-Rural Comparison Using GPS 

**DOI:** 10.3390/ijerph9093030

**Published:** 2012-08-24

**Authors:** Peter Collins, Yahya Al-Nakeeb, Alan Nevill, Mark Lyons

**Affiliations:** 1 School of Human Sciences, Newman University College, Genners Lane, Bartley Green, Birmingham B32 3NT, UK; Email: p.collins@newman.ac.uk; 2 School of Performing Arts and Leisure, University of Wolverhampton, Walsall Campus, Gorway Road, Walsall WS1 3BD, UK; Email: a.m.nevill@wlv.ac.uk; 3 Faculty of Education and Health Sciences, University of Limerick, Limerick, IRL, Ireland; Email: mark.lyons@ul.ie

**Keywords:** free-living physical activity, adolescent, Global Positioning System

## Abstract

The built environment in which young people live has a significant influence on their physical activity (PA). However, little is known regarding how youth from suburban and rural settings utilise their surrounding environments to participate in free-living PA. 50 adolescents aged 13–14 years old (22 rural; 28 suburban) wore an integrated GPS and heart rate device during non-school hours and completed a daily PA diary over 7 days. Descriptive statistics and analyses of variance were used to explore differences in the amount and location of moderate-to-vigorous physical activity (MVPA) between genders and youth from different geographical settings. Suburban youth participated in significantly (*p* = 0.004) more daily PA (52.14 minutes MVPA) and were more extensive in their utilisation of their surroundings, compared to rural youth (26.61 minutes MVPA). Suburban youth visited more public recreational facilities and spent significantly more time outdoors and on local streets (109.71 minutes and 44.62 minutes, respectively) compared to rural youth (55.98 minutes and 17.15 minutes, respectively) during weekdays. Rural youth on average spent significantly more time within the home (350.69 minutes) during weekends compared to suburban youth (214.82 minutes). Rural females were the least active group of adolescents, participating in the least amount of daily PA (20.14 minutes MVPA) and spending the least amount of time outdoors (31.37 minutes) during weekdays. Time spent outdoors was positively associated with PA. The findings highlight the disparity in PA levels and the utilisation of the surrounding built environment between youth from two different geographical settings and possible environmental causes are discussed. The study supports the use of GPS (combined with other methods) in investigating geographical differences in young people’s PA and movement patterns. This method provides a wealth of information that may assist future policies and interventions in identifying environmental characteristics that promote PA in youth from different geographical settings.

## 1. Introduction

The advantages of physical activity (PA) to people of all ages are widely publicised and present psychological, sociological and physiological benefits to those who regularly engage in PA [1,2,3]. The health risks of a sedentary and inactive lifestyle are also undeniably clear, with physical inactivity accounting for approximately 6 percent of deaths globally, making it the fourth leading risk factor for global mortality [4]. Furthermore, PA provides the backbone of total energy expenditure and it is widely acknowledged that in order for people to attain a healthy weight status they must ensure that they achieve a balance between energy intake and energy output [5,6]. Statistics in the UK replicate many other countries globally indicating that only a small percentage of young people meet the recommended PA guidelines for health [7,8]. 

In order to promote PA in youth it is important that young people are provided with the opportunities to be active within their surrounding social and physical environment. Many people in today’s society live in obesogenic environments which play a key role in promoting weight gain by encouraging inactivity or poor diet (overeating or consumption of unhealthy foods) [9]. Swinburn *et al*. [10] stated that obesity is the outcome of people responding normally to the surrounding obesogenic environment in which they live. Whilst significant advances have been made in exploring the influence of the built environment (BE), researchers are still searching to clearly outline the identity and impact of specific BE characteristics on the PA patterns and health habits of young people. This has caused attempts to create effective strategies and policies which provide PA supportive environments to be both troublesome and generally inefficient. Previous literature has clearly shown that environmental influences on young people differ significantly depending on demographic factors such as age, gender, socioeconomic status, race and ethnicity [11,12,13]. Subsequently, a ‘one size fits all’ blanket approach to promoting PA which assumes that ‘background’ components are equal [14] amongst all youth is too simplistic and vague, and offers little or no benefit to many young people’s health and lifestyles.

In addition to demographic factors such as age, gender, and socioeconomic status, the geographical setting in which young people live can also play a major role in establishing the influence of specific BE characteristics on a young person’s PA patterns and health habits. This is due to the young people living in different geographical settings interacting, perceiving and utilising their surrounding BE differently for free-living PA purposes, with previous literature outlining significant differences in the PA patterns of urban and rural youth [15,16,17]. However, the direction of PA level disparities is not consistent between these studies, whilst other study findings revealed no difference in PA amount [18,19]. A review by Sandercock *et al*. [18] concluded that given the vast disparity between rural and urban settings, it is perhaps unsurprising that previous literature has provided ambivalent findings regarding the PA levels of youth from these contrasting environments. It was therefore, reasoned that the suburban environment may be the most conducive for young people to be physically active, as it lacks many of the extreme barriers that either a rural or urban landscape include. For example, whilst urban dwelling youth may face high traffic density and rural youth may face high commuting distances or poor infrastructure, such PA barriers could be potentially less extenuated in a suburban environment.

The difficulties in exploring the influence of the BE on young people’s free-living PA patterns may also be attributed to the limitations of traditional measures of PA, with the vast majority of previous literature relying upon subjective measures to record young people’s locations during their free-time. However, the relatively recent emergence of Global Positioning Systems (GPS) provides an accurate and objective measure of the location of PA. It also enables the researcher to gain a greater insight into how young people utilise their surrounding BE to be physically active, and identify the environmental facilitators and barriers that different geographical environments present to young people. Initial research utilising GPS has highlighted its ability to measure young people’s PA in numerous settings such as the school playground [20,21,22], the commuting journey to school [23], public playgrounds [24] and the general neighbourhood environment [25,26]. Whilst the utilisation of GPS provides a wealth of data, it is widely acknowledged that GPS is most effective when combined with other measures of PA [27], with either heart rate monitors [20,21,22] or accelerometers [23,24,25,28] most commonly used. Furthermore, previous studies using GPS also used [26] or recommended [25] the use of a qualitative activity diary. A mixed methods approach can provide a detailed insight into young people’s PA patterns, and allows the researcher to establish when and why young people did not wear the device and what they were doing when it was not worn. 

A study by Jones *et al*. [26] utilised GPS to reveal significant differences in the manner in which younger children (9–10 years) used their surrounding BE to be active and two studies by Fjortoft *et al*. [20,21] also reveal intriguing findings regarding how young people interact with their surrounding school playground environment to be active during recess. However, sparse previous literature has explored the free-living PA patterns of adolescent aged youth from differing geographical environments over a 7 day period utilising GPS. Whilst a study by Quigg *et al*. [24] used GPS to measure PA in young children over 7 days, further studies of adolescent aged youth over a whole week period are scantly available. Moreover, the most conducive environment for PA as highlighted in a review by Sandercock *et al*. [18] may be the suburban environment, which has been under studied in previous literature [29].

Given the relatively recent introduction of GPS as a viable method of measuring free-living PA, limited research has explored geographical differences in UK adolescents’ free-living PA. Therefore, the aim of the current study was to explore the use of GPS combined with heart rate monitoring in objectively measuring the PA patterns of adolescent aged youth in contrasting geographical environments over a seven day period. 

## 2. Methods

### 2.1. Participants

The study was conducted in Central England, with the rural based school located in South Staffordshire and the suburban based schools in the West Midlands and South Staffordshire. In total, 50 adolescent 13–14 year olds including 22 rural (10 male and 12 female) and 28 suburban (12 male and 16 female) youth agreed to participate in the study by providing informed and written personal and parental consent. Three participants from both the rural and suburban schools did not provide sufficiently complete data, resulting in a final sample size of 44 participants (25 females and 19 males). Rural participants attended a rural village school whilst suburban participants were from two suburban schools. The rural school is situated in a village within the South Staffordshire countryside and lies at the foot of a range of hills [30]. Within this rural setting recreational provisions are provided in the form of a local leisure centre (open during evenings on the same site as the High school) and a nearby country park. Numerous trekking trails are accessible from the village into the surrounding countryside and green belt area. However, there are no public parks, playing fields or playgrounds within the village itself or the surrounding countryside. It should also be acknowledged that the catchment area for the school is large and encompasses students from other nearby rural villages, the city of Wolverhampton (which is approximately 11 miles away) and the large conurbation area of the West Midlands. 

The suburban schools are situated in the suburbs of the City of Wolverhampton and have been described as urban-fringe or small town settlements. They are approximately 4 miles away from Wolverhampton city centre [30] and numerous local public recreational facilities are available within these built environments, including leisure centres, numerous local parks, playing fields and playgrounds, gymnasiums, a nature reserve and nearby golf and cricket clubs. Both suburban schools were in close proximity to each other (3.3 miles) and shared very similar socio-demographic features (e.g., socioeconomic status, race/ethnicity, and suburban environment). All of the schools were of similar socioeconomic and race/ethnicity background whilst the geographic locations of the schools were determined from census data, Ofsted reports and using a Geographical Information System (GIS) to identify specific facilities within each environment. The socioeconomic status of each participatory school was established via the school’s latest Ofsted report and percentage of children eligible for free school meals.

### 2.2. Measures

PA was measured using a GPS device (Garmin Forerunner 305, Garmin Ltd., Olathe, KS, USA) and subjective PA diary. The GPS monitor enabled the objective measurement of the participants’ location, speed, distance travelled, elevation, pace, and calories expended. The Garmin Forerunner 305 GPS device is synchronised and wirelessly connected with a heart rate monitor which participants wear around the chest, to provide an additional indication of PA intensity. Previous literature has supported the feasibility and validity of this method in measuring young people’s PA [20,21,22,25,26]. In the current study, the participants wore the GPS monitor on their wrist. This was standardised in light of previous literature highlighting reduced accuracy of the monitors when they are worn either in a lanyard or around the waist [31]. The GPS device was set on a ‘smart recording’ setting so that data points were recorded whenever there was a significant change in the participants’ movements (*i.e.*, speed, direction, elevation, distance) or heart rate intensity. This ensured that battery life and memory capacity were sufficient to record daily free-living PA. 

An adapted version of the Heyward [32] PA diary was utilised to subjectively record PA. Participants were required to complete the activity diary during the evening of each day, and for any activity recalled, the participant was requested to provide the following details; type of activity, intensity of activity, time spent on activity, and whether they wore the GPS during the activity. Previous research supports the use of activity diaries such as this as a measure of PA in adolescents [33] Examples were provided on the activity diary for the ‘intensity of activity’ question (e.g., very tiring, tiring, easy) to ensure that all participants understood how to accurately complete the diary. This provided data that can be used for triangulation purposes, to cross compare with the GPS findings and ensure concurrent validity between the quantitative and qualitative measures of PA [34,35]. Furthermore, the activities that the participants were involved in when they were not wearing the GPS device could also be recorded, as recommended in previous studies [25]. Participants’ height and weight were recorded (without shoes or excess clothing) to calculate Body Mass Index (BMI). Height was measured to the nearest 0.5 centimetre using a Seca portable height measure (Seca Ltd., Hamburg, Germany) and weight was measured to the nearest 100 grams using Seca weight scales (Seca Ltd., Hamburg, Germany). In accordance with the International Obesity Task Force (IOTF) criteria, age and gender specific BMI cut-off points were used to establish obesity classification [36]. 

### 2.3. Procedure

Participants were asked to wear the GPS device and heart rate monitor during their free-time (out-of-school hours) for seven days (Monday-Sunday) and testing commenced after Institutional Ethical Approval was granted and the necessary parental and participant consent had been received. Due to the study aims, the adolescent’s activities were only recorded during their free-time (out-of-school hours), which consisted of their waking hours between the end of the school day and the beginning of the following school day. On average, the duration of time that adolescents were expected to wear the device was approximately 10–12 hours per day for up to a maximum of 7 days. Due to the restricted memory storage capacity and battery life (approximately 10–12 hours) of the GPS devices, adolescents were required to return the devices to the researcher on a daily basis (each morning when arriving at school) and collect the devices at the end of each school day. This provided the researcher with sufficient time to upload all of the participants’ GPS data, clear the memory and fully recharge all of the GPS watches. This procedure minimised participant burden during the school week and enabled the researcher to check daily that the research procedure was being adhered to (*i.e.*, participants were wearing the device, completing the activity diary and the GPS devices were in working order). During the weekend, the participants received verbal training and written instructions on how to recharge the GPS device, so that they could wear the GPS device throughout the weekend period. The reliance on adolescent-aged participants to recharge the device is supported by previous literature which also adopted this approach [25].

All participants received detailed instructions about how and when to complete the activity diary and wear the GPS and heart rate monitors. These instructions and measurements were conducted during school hours. Issues regarding data recording and data accuracy during the GPS initialisation period were minimised by the researcher instructing all participants to turn on the GPS devices when they were stationary in an outdoor environment, as recommended in previous research [31]. 

### 2.4. Data Processing and Statistical Analysis

A range of statistical procedures were performed on the data to establish associations and differences in the PA patterns of suburban and rural youth. Comparisons between the different geographical locations and genders were conducted using two-way analysis of variance (ANOVA) on the young people’s PA levels, time spent in specific locations and BMI. A two-tailed significance value of *p* < 0.05 was considered significant in all statistical analysis. Descriptive statistics were utilised to highlight the prevalence of overweight and obesity as well as the proportion of youth who achieved the recommended daily PA guidelines of 60 minutes of moderate-to-vigorous physical activity (MVPA) [37]. Furthermore, Pearson correlations were performed to establish relationships between behavioural patterns and health status variables (e.g., time spent in the house, BMI and PA levels). SPSS version 19 (SPSS Inc., Chicago, IL, USA) was used for all analyses. 

A mathematical technique called trilateration is used to calculate the GPS information (such as location, elevation and speed) and details regarding the recording and analysis of GPS data have been provided in a previous review [27]. Raw GPS location data was extracted from the devices using GPS-Utility software before subsequent analysis using the Garmin training centre and Garmin Connect programs (Garmin Ltd., Olathe, KS, USA). The data was then divided based on both the participants location (e.g., in the house or on the street) and heart rate determined PA level (e.g., inactive, MVPA or vigorous PA) [20]. For example, all of the time that youth were recorded via the GPS as being located in a specific environment (such as in a public park) was extracted and independently analysed to establish activity levels within the designated environment. This allowed for movement patterns to be established based on analysing both where young people went during their free-time and whether these environments promoted PA. The time that young people spent in an automobile or indoors (such as in a house, shop or sports centre) was excluded from the analysis of time spent outdoors. Therefore, time outdoors is based solely on the time that youth were outside on foot (or on a bicycle). PA intensity was determined from the findings of the heart rate monitor and when applicable the GPS data (e.g., speed). PA cut-off points were set at <120 bpm (inactive), 120–139 bpm (low to moderate), 140–159 bpm (moderate to vigorous) and >160 bpm (vigorous) as performed in previous research [21] with total MVPA calculated as total time (in minutes) spent being active. By combining both objective methods and the subjective activity diary, the lead researcher could accurately interpret when each participant was participating in bouts of PA. 

For inclusion in the data analyses, adolescents had to provide a minimum of 3 hours of full GPS and heart rate data for at least one day [28]. Therefore, given the likelihood of differing numbers of days completed by each participant (dependent on participant compliance), the mean daily PA data was stacked and weighted in accordance to the number of days that each participant provided at least 3 hours of full data. Thus, all statistical analysis conducted on the data collected via the GPS and heart rate monitors is based on the weighted statistics, in order for the findings to provide a fairer reflection on the average daily PA levels of participants. The weighting of statistical data in this manner is fully discussed in previous literature [38].

## 3. Results

### 3.1. Participants

Of the 50 adolescents that agreed to participate in the study, three participants from the rural school and three from the suburban schools did not provide usable data, resulting in 88% of participants providing data which contributed to the outcome of the study (44 participants; 25 females and 19 males). From the total number of participants who provided analysed data, the overall average number of days that participants wore the GPS monitor was 4.23 (±1.72) days per week, whilst the average time they wore the device per day was 6.46 (±1.50) hours. 

**Table 1:** Mean (±SD) BMI and PA across the whole week, weekdays and weekend.

Variable	Suburban		Rural	
	Male *n* = 12	Female *n* = 16	Male *n* = 10	Female *n* = 12
BMI (kg/m^2^) *	23.22 ± 5.46	19.99 ± 2.71	21.16 ± 3.03	23.22 ± 5.80
**Whole week PA levels**				
Daily MVPA (mins) **	49.90 ± 59.90	53.99 ± 41.15	36.17 ± 57.69	18.20 ± 37.25
Daily VPA (mins) **	21.03 ± 32.06	16.96 ± 21.72	12.18 ± 23.94	3.98 ± 8.21
**Percentage of subgroup that met 60 mins MVPA guidelines**	36.4%	28.6%	12.5%	9.1%
**Weekday PA levels**				
Daily MVPA (mins) **	56.13 ± 55.46	56.43 ± 51.79	31.28 ± 58.24	20.14 ± 55.31
Daily VPA (mins) **	24.01 ± 30.17	16.68 ± 25.58	6.84 ± 12.56	4.60 ± 9.31
**Percentage of subgroup that met 60 mins MVPA guidelines**	27.3%	28.6%	25%	10%
**Weekend PA levels**				
Daily MVPA (mins) *	26.56 ± 28.78	53.16 ± 37.38	49.00 ± 62.64	13.03 ± 18.69
Daily VPA (mins) *	7.36 ± 12.92	17.75 ± 21.04	26.22 ± 50.57	2.32 ± 5.57
**Percentage of subgroup that met 60 mins MVPA guidelines**	16.7%	40%	20%	0%

* Statistically significant interaction between suburban males and rural females with rural males and suburban females (*p* < 0.05); ** Statistical significant difference between rural and suburban youth (*p* < 0.01).

There was no significant difference in BMI across genders or geographic locations (*p* > 0.05). Despite these findings, a statistically significant interaction in BMI between gender and geographical setting was apparent, with suburban males and rural females having a significantly higher BMI compared to both rural males and suburban females (see Table 1). However, a Chi square analysis of independence revealed no significant difference in the obesity classifications of suburban and rural youth (*p* > 0.05), with approximately one in four adolescents being overweight or obese in both the rural and suburban settings. A Pearson correlation analysis also revealed no significant relationship between BMI and either PA level, PA intensity, level of compliance (based on number of days completed or time the device was worn) or mode of transport when commuting home from school (*p* > 0.05). 

### 3.2. Whole Week PA Level

**Figure 1:**
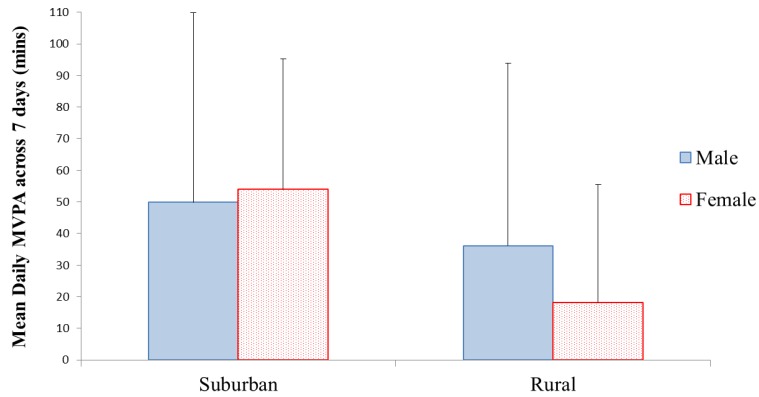
Mean (±SD) daily PA of rural and suburban youth across the 7 days.

There was a significant difference in the average daily MVPA levels of all rural and all suburban youth over the course of the week (F_(1,43)_ = 9.147, *p =* 0.004), with suburban youth participating in significantly more daily PA over the 7 days (52.14 minutes MVPA) compared to rural youth (26.61 minutes MVPA) (see Figure 1). In the UK, current health guidelines recommend that youth should engage in 60 minutes of daily MVPA [37] and the disparity between suburban and rural youth is highlighted by 90% of rural youth not meeting these PA guidelines, compared to 68% of suburban youth. Suburban youth also participated in significantly more VPA (F_(1,43)_ = 8.006, *p =* 0.007) compared to their rural counterparts (18.80 minutes and 7.82 minutes average daily VPA, respectively). These findings indicate the differences between the overall group (males and females combined) of rural and suburban youth. However, when analysing the genders independently differences in PA levels of suburban and rural youth were especially evident when comparing females rather than males (*p* > 0.05). This is illustrated in Table 1. However, there were no significant differences when comparing males and females directly, either within or between the different geographical settings.

When subdividing the overall findings into weekday and weekend findings; the weekday PA levels replicated the whole week findings, with suburban youth participating in significantly more MVPA (F_(1,42)_ = 8.203, *p =* 0.007) and VPA (F_(1,42)_ = 11.267, *p =* 0.002) compared to rural youth. During weekends however, there was no significant difference in MVPA or VPA between suburban and rural youth (*p* > 0.05). A significant interaction in MVPA was apparent however, with the two-way ANOVA indicating that in the suburban setting females were the most active, whereas males in the rural setting were more active. Table 1 illustrates similarities and differences between geographical and gender subgroups for weekday and weekend PA levels. As highlighted in Table 1, rural females throughout the 7 day testing period were consistently the least active subgroup, with not one rural female participating in sufficient MVPA during the weekend. 

When comparing weekday and weekend PA levels of youth, there were no significant differences in average MVPA or VPA (*p* > 0.05). A Pearson correlation also revealed no significant relationship between weekday and weekend PA levels (*p* > 0.05). Furthermore, there was no significant difference in the time that youth spent in locations such as the home, recreational facilities or outdoors between the weekdays and the weekend (*p* > 0.05). Even when accounting for gender and geographical setting differences, no statistically significant differences were apparent. 

### 3.3. PA Patterns and the Utilisation of the Surrounding BE

The utilisation of GPS and heart rate monitoring allows for not only the identification of differences in the amount and intensity of PA between different demographic groups of youth, but it also objectively measures the nature of PA patterns, with regards to the location and time spent participating in PA within the BE. The participants visited a broad range of locations throughout the seven days of testing. However, many of these locations were visited infrequently or by a small minority of the sample. In order to establish whether these environments were conducive to PA in youth, they had to be clearly identified. Throughout the data analysis stage, the GPS and heart rate findings were supplemented with the subjective activity diary information provided by the participants. This information was integral in clearly establishing not only the location and intensity of activity, but also the type of activity. Furthermore, on occasions participants entered buildings which were unidentifiable using GIS or GPS data alone. However, the activity diaries provided clarity on what these buildings were and furthermore, whether they were being utilised for PA. Subsequently, the findings of the results which follow pertaining to specific environments are based on a combined mixed methods approach. 

#### 3.3.1. Time Outdoors

**Figure 2:**
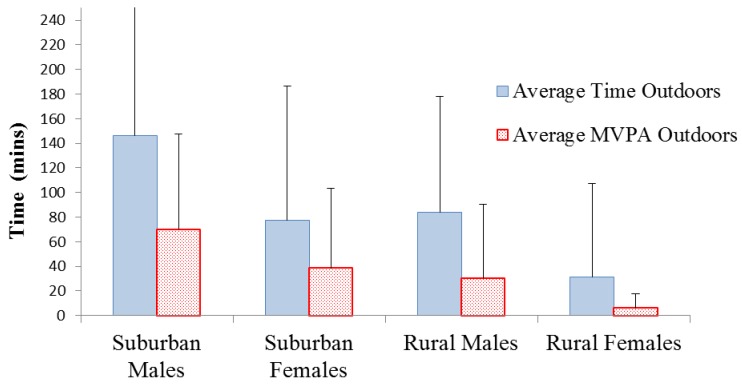
Weekday mean (±SD) outdoor time and physical activity of suburban and rural youth.

A Pearson correlation revealed that the time youth spent outdoors was positively associated with MVPA during both the weekdays (r = 0.420, *p =* 0.005) and the weekends (r = 0.550, *p =* 0.004). Furthermore, a two-way ANOVA (F_(1,42)_ = 6.181, *p =* 0.017) revealed that suburban youth spent significantly more time outdoors during weekdays compared to rural youth (109.71 minutes and 55.98 minutes respectively). Descriptive statistics indicate a similar pattern during weekends, however the difference between the time spent outdoors between suburban (93.22 minutes) and rural (51.74 minutes) youth was not statistically significant (*p* > 0.05). During both the weekdays (F_(1,42)_ = 9.616, *p =* 0.004) and the weekend (F_(1,25)_ = 4.662, *p =* 0.042), suburban youth participated in significantly more MVPA outdoors and in their surrounding BE (see Table 2). For example, during the weekend suburban youth participated in 38.59 minutes of daily MVPA outdoors, whilst rural youth participated in just 6.72 minutes of MVPA outdoors. Gender differences were only apparent during the weekday results (F_(1,42)_ = 7.779, *p =* 0.008), with suburban and rural males spending significantly more time outdoors (146.19 minutes and 84.11 minutes, respectively), compared to suburban and rural females (77.52 minutes and 31.37 minutes, respectively), as shown in Figure 2. On average, males spent 37.73% of their free-time outdoors during weekdays compared to females spending 18.68%. 

**Table 2:** Weekday mean (±SD) PA and behavioural patterns of youth within different environments.

Variable	SUBURBAN		RURAL	
	Male * n* = 12	Female * n* = 16	Male * n* = 10	Female * n* = 12
**Time spent outdoors (mins)** *	146.19 ± 147.80	77.52 ± 108.67	84.11 ± 94.18	31.37 ± 76.02
**Outdoor daily MVPA (mins)** **	70.35 ± 77.11	38.75 ± 64.52	30.41 ± 59.76	6.31 ± 11.45
**Outdoor daily VPA (mins)** **	12.08 ± 4.72	4.72 ± 10.42	1.88 ± 4.43	0.09 ± 0.27
**Percentage of subgroup that spent time outdoors**	100%	100%	100%	90.91%
**Time spent in public recreational facilities (mins)**	99.36 ± 122.25	50.00 ± 75.58	48.35 ± 116.84	30.00 ± 84.29
**Public recreational facilities daily MVPA (mins)** *	56.40 ± 72.83	30.22 ± 56.31	18.88 ± 61.65	11.22 ± 31.89
**Public recreational facilities daily VPA (mins)** *	11.51 ± 24.08	4.86 ± 12.41	1.03 ± 2.60	1.35 ± 4.94
**Percentage of subgroup that visited recreational facilities**	100%	100%	100%	90.91%
**Time spent in the house (mins)**	218.42 ± 143.50	216.21 ± 114.76	268.94 ± 94.97	246.33 ± 111.10
**House daily MVPA (mins)**	8.28 ± 10.57	18.27 ± 23.35	8.35 ± 14.14	9.78 ± 51.91
**House daily VPA (mins)**	0.07 ± 0.19	0.74 ± 4.25	0.32 ± 0.81	0.39 ± 1.11
**Percentage of subgroup that spent time in the house**	100%	100%	90.91%	90.91%
**Time spent on the street (mins)** **	47.90 ± 43.68	41.56 ± 41.69	14.70 ± 25.56	19.94 ± 14.37
**Street daily MVPA (mins)**	15.48 ± 11.29	16.93 ± 18.44	12.77 ± 37.77	6.13 ± 8.83
**Street daily VPA (mins)**	0.63 ± 1.26	0.68 ± 1.48	0.99 ± 4.99	0.14 ± 0.32
**Percentage of subgroup that visited the street**	72.73%	78.57%	75%	54.55%
**Time spent in automobiles (car/bus) (mins)**	18.31 ± 21.59	13.98 ± 29.04	18.62 ± 10.19	26.43 ± 37.59
**Automobile daily MVPA (mins)**	0.57 ± 2.44	1.37 ± 3.79	0.49 ± 0.18	0.33 ± 0.11
**Automobile daily VPA (mins)**	0 ± 0	0 ± 0	0 ± 0	0 ± 0
**Percentage of subgroup that spent time in automobiles**	54.55%	71.43%	87.5%	63.64%

* Statistical significant difference between rural and suburban youth (*p* < 0.05); ** Statistical significant difference between rural and suburban youth (*p* < 0.01).

#### 3.3.2. Time Indoors

Whilst the results indicated that spending time outdoors was positively associated with PA levels in youth, it was also revealed that percentage of free-time spent indoors (within the home) is negatively associated with MVPA during weekends (r = −0.523, *p =* 0.006) and with VPA during weekdays (r = −0.417, *p =* 0.006). Furthermore, the weekend results also revealed a significant difference in the time spent indoors (within the home) between youth from different geographical settings (F_(1,25)_ = 7.874, *p =* 0.010) with suburban youth (214.82 minutes) spending significantly less time in the house compared to rural youth (350.69 minutes). Whilst the difference between suburban and rural youth’s PA levels during weekdays was not statistically significant, the descriptive statistics again indicate that suburban youth spent less time indoors compared to rural youth (see Table 2). The negative association between time indoors and PA was underlined with active youth (who met the recommended 60 minutes of MVPA guidelines) spending a significantly lower proportion of their free-time in the home compared to less active youth (who did not achieve the recommended PA level guidelines) during both the weekdays (F_(1,42)_ = 6.621, *p =* 0.014) and the weekend (F_(1,25)_ = 5.326, *p =* 0.030). For example, ‘active’ youth spent 57.16% of their free-time during weekdays and 46.60% of free-time during the weekend indoors, compared to ‘less active’ youth spending a significantly higher proportion of free-time indoors (74.74% and 76.76% respectively). This equated to ‘active’ youth spending only half of their free-time in the house, compared to ‘less active’ youth spending approximately three-quarters of their free-time in the house.

#### 3.3.3. Street Environment

A Pearson correlation revealed a significant positive association between the time youth spend on the street and their VPA level during the weekdays (r = 0.438, *p =* 0.014). With this in mind, a univariate ANOVA also revealed a significant difference in the time that rural and suburban youth spend on local neighbourhood streets during the weekdays (F_(1,30)_ = 11.758, *p =* 0.002). On average, suburban youth spent 44.62 minutes of each school day on the streets of their local neighbourhood environment, compared to rural youth who spent only 17.15 minutes. This may be due to differences in commuting patterns, as 78.6% of suburban youth walked home from school, compared to just 36.4% of rural youth who actively commuted. Such differences have contributed to approximately a third of rural youth not spending any time outside on foot (or bike) in a local neighbourhood street environment, compared to a quarter of suburban youth not visiting a street environment during school days. Similar findings are presented when analysing weekend behavioural patterns (F_(1,13)_ = 6.724, *p =* 0.027), as suburban youth spent a significantly higher proportion of their free-time on a local street compared to rural youth (12.78% and 3.36% of free-time, respectively). 

**Figure 3:**
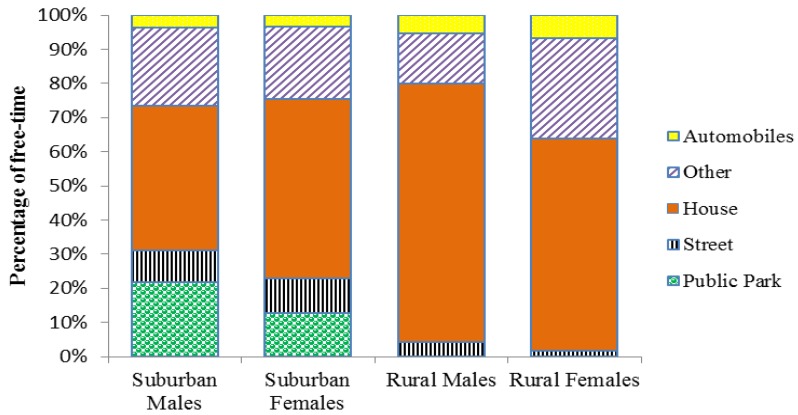
Percentage of free-time that suburban and rural youth spend in different environments on average during weekdays.

In terms of time spent in an automobile, there was no significant difference in the PA levels of rural and suburban youth (*p* > 0.05). However, a univariate ANOVA did reveal that for youth who travelled by automobile during the weekend, females spent significantly more time in automobile transport compared to males (F_(1,17)_ = 5.595, *p* = 0.033), regardless of geographical location. The same significant difference was also apparent when comparing percentage of weekend free-time spent in a car or bus between genders (F_(1,17)_ = 6.605, *p* = 0.022). Whilst a similar percentage of males and females utilised automobile transport during the weekend (73% and 67%, respectively), females on average spent 44.28 minutes in an automobile being transported compared to males spending just 19.34 minutes. Weekday differences in the proportion of free-time that youth spend in different environments such as the street, park and indoors are illustrated in Figure 3.

#### 3.3.4. Local Recreational Facilities

When focusing specifically on how youth utilised local public recreational amenities such as parks, public playgrounds, leisure centres, school playing fields and country parks, a univariate ANOVA revealed a significant difference in suburban and rural youth’s MVPA when in public recreational facilities. On weekdays, when young people visited these recreational facilities, suburban youth participated in significantly more MVPA (F_(1,42)_ = 6.301, *p* = 0.016) and VPA (F_(1,42)_ = 6.221, *p* = 0.017) compared to their rural counterparts. This is highlighted by the descriptive statistics in Table 2. Moreover, youth who met the recommended PA guidelines of 60 minutes of MVPA per day spent significantly more time (F_(1,42)_ = 10.464, *p* = 0.002) at these recreational facilities compared to youth who did not meet the recommended PA guidelines (111.05 minutes and 42.51 minutes, respectively). Whilst a significant proportion of suburban youth visited several recreational amenities, not one young person from a rural setting visited a public park, playing field or playground during either the weekdays or weekend. This may have contributed to overall findings revealing that suburban youth are more active than rural youth; for example, approximately one in three suburban youth visited a playing field during the weekdays and on average participated in 49.63 minutes of MVPA. Furthermore, these findings reflect the disparity in the provisions of recreational amenities between the suburban and rural environments, with the rural village possessing numerous green spaces, open country fields, walking trails, a public leisure centre and a country park, but no designated public parks, playgrounds, or marked playing fields. This is in contrast to the suburban environment which possessed numerous playing fields (with marked football pitches), public parks and playgrounds. 

There was no statistical difference in the public playground MVPA levels between suburban males and females, although descriptive statistics indicated that females participated in more MVPA in the playground (22.27 minutes) compared to males (3.29 minutes). In contrast, within the playing field environment, suburban males participated in more MVPA on an average weekday (59.35 minutes) compared to suburban females (6.88 minutes), although this difference was not statistically significant (*p* > 0.05). 

The GPS identified an array of other environments which youth visited throughout the week such as shops, restaurants, cinemas, theatre, garden, woodland and extra-curricular education classes. However, such environments were scarcely visited and did not significantly impact upon the results of the study.

## 4. Discussion

The study explored the use of GPS in investigating the PA behavioural patterns of a sample of adolescent suburban and rural youth. The findings underlined the ability of GPS (when combined with a heart rate monitor and subjective daily activity diary) to clearly measure and identify differences in PA patterns in youth from different geographical environments. The study revealed that suburban youth were more active than their rural counterparts, as indicated in previous literature [18]. Whilst geographical differences were evident, the PA levels of the different sexes did not significantly differ, with a higher proportion of both suburban males and females (36.4% and 28.6%, respectively) meeting the recommended guidelines of 60 minutes MVPA per day [37], compared to rural males and females (12.5% and 9.1%, respectively). The interactions and movement patterns of suburban and rural youth, within their surrounding BE, differed significantly. 

The importance of youth spending time outdoors is apparent throughout the study findings, irrespective of geographical setting, gender or stage of the week (weekends or weekdays). This was highlighted by the positive association between time spent outdoors and PA level, supporting previous study findings utilising GPS [26,28] and other methods [39,40]. This indicates that regardless of whether the surrounding BE was a suburban or rural setting, when young people were outdoors they were more likely to be physically active. However, this does not depreciate the role of the BE and the importance of providing accessibility and amenities for PA, as without a PA promoting environment youth will not have the incentives or reasons to spend their free-time outdoors. This is highlighted in the study findings with suburban youth on average participating in significantly more PA across the whole week. With the suburban environment offering a broader range of recreational facilities such as public parks, playing fields and playgrounds it can be argued that the suburban environment contained more attractions to encourage youth to spend more time outdoors and within their surrounding environment. It is therefore imperative, given the growing body of evidence, that future government and land-use policies adapt and enhance the neighbourhood BE to encourage youth to spend more time outdoors, predominantly through the provision of safe and PA supportive environments. 

The time that youth spent indoors was also associated with PA level, with active youth spending significantly less time in the home compared to less active youth. Previous literature has highlighted the numerous opportunities for sedentary behaviour within the home environment, with sedentary activities such as television viewing and computer gaming having a negative association with health and obesity [41,42,43]. In addition, previous research trends have indicated that young people are spending less time outdoors in their surrounding neighbourhood environment [44,45], and more time participating in sedentary activities such as watching television or playing on a computer compared to previous generations [46]. The current study concurs with previous literature in underlining the importance of encouraging youth to spend more of their free-time outdoors and away from the home environment. 

The results not only indicated that suburban youth spent less time within the home and more of their free-time outdoors, but also demonstrated suburban youth’s broader utilisation of the surrounding BE in which they lived. Suburban youth’s broader use of their environment could be partially due to the broader range of recreational amenities that they have access to within their surrounding BE. However, this would not explain the greater use of some environments which were equally accessible to both rural and suburban youth. For example, suburban youth spent significantly more time than rural youth on the streets of their local neighbourhood environment during both weekdays and the weekend. On weekdays suburban youth spent on average 44.62 minutes on the street compared to rural youth who spent just 17.15 minutes outdoors on the streets of the local environment. Whilst both rural and suburban youth have access to the street environment, infrastructural characteristics of the suburban streets may be more conducive to PA, and subsequently encourage suburban youth to spend more time in this environment. The influence of street infrastructure is highlighted by previous literature that indicate that street lighting, well maintained sidewalks, high street interconnectivity and the presence of cul-de-sacs are all potential promoters of PA in youth [47,48,49]. Such characteristics were substantially more evident in the suburban environment compared to the rural environment. 

School commuting patterns may have also contributed to the disparity in time spent on the streets between suburban and rural youth, with 78.6% of suburban youth actively commuting home from school compared to just 36.7% of rural youth. Rural youth on average commuted a greater distance compared to suburban youth (3.11 miles and 1.20 miles, respectively), and these findings are perhaps unsurprising, given previous literature highlighting the commute distance as a primary determinant of commute choice in youth [50,51,52]. However, this factor could not explain the difference in time spent on the street during the weekend and this further supports the notion that with the suburban environment possessing greater street connectivity and greater access to recreational amenities, this may have encouraged youth to spend more time outdoors. 

Not a single adolescent in the rural setting visited a PA supportive public recreational facility such as a park, playground or playing field over the 7 day testing period. In contrast, over one third of suburban youth visited one of these environments at least once during the weekday testing period. This highlights the disparity in suburban and rural youth’s utilisation of public recreational facilities such as parks, playgrounds and playing fields. With previous literature highlighting the positive association between these public amenities and PA or health [53,54,55,56,57] combined with the current study highlighting the importance of these environments in assisting youth in attaining 60 minutes of MVPA each day, it is extremely concerning that none of the rural youth visited a park or playground environment over the 7 day testing period. This provides further explanation for the disparity in PA levels between suburban and rural youth, and supports previous literature [58] in highlighting the importance of the provision of local recreational amenities. With the rural environment not containing any public parks, playing fields or playgrounds it is perhaps unsurprising that no rural youth visited such facilities as their access to such amenities was substantially hindered. However, it should not be overlooked that other facilities such as trekking trails, open fields, and a leisure centre were all present in the rural setting. This may indicate that these environments whilst having the potential to promote PA, were perceived as too costly (in the case of the leisure centre) or as being an insufficient incentive to attract the adolescents to commit their free-time to frequently visiting these locations. These tentative findings emphasise the importance of differentiating between different types of recreational facilities when assessing their potential impact on young people’s PA. 

Whilst significant gender differences in PA levels were not apparent, differences in the behavioural patterns of the different sexes were occasionally evident. For example, during the weekend females spent significantly longer periods in automobiles compared to males (44.28 minutes and 19.34 minutes, respectively), whilst females spent significantly less time outdoors during weekdays compared to males. Previous literature has indicated that parents are often more protective and safety cautious over their young females compared to young males [59,60]. This may have contributed to the current findings, with parental influence resulting in young males being allowed more independence and freedom to explore and actively commute within their surrounding neighbourhood compared to young females, who are perceived as more vulnerable [61]. Despite these findings, across all the analyses of PA and movement patterns, rural females were consistently and overwhelmingly the least active group of young people, in terms of their overall PA levels and their utilisation of the surrounding BE. For example, rural females visited the least number of recreational facilities, spent the least amount of time outdoors, and spent the highest percentage of free-time in the home. In supporting previous findings [62], the current study can therefore underline the importance of targeting young rural females in future research and policies aimed at tackling inactivity and promoting healthy living.

The current study provided an insight into the measurement of young people’s free-living PA patterns by utilising GPS over a seven day period, a methodology scarcely explored by previous researchers. Furthermore, the utilisation of a daily PA diary to supplement the GPS and heart rate monitor findings greatly assisted the researchers in establishing clear patterns of PA and in identifying specific locations of PA. This mixed methods approach provided a detailed insight into the free-living PA patterns of young people in their surrounding BE and future research should consider including this subjective measure when using GPS and other objective methods of measuring PA. 

Whilst the seven day testing period ensured that 88% of participants provided at least one day of acceptable data for analysis, the relatively high research burden caused non-compliance rates to rapidly increase towards the latter stages of the 7 day testing period, with only 58% of these participants providing sufficient data for testing days 6 or 7 and participants providing on average 4.23 days of analysed data across the 7 day testing period. This is understandable, given the high research participant burden involved with wearing the GPS and heart rate monitoring devices and completing a daily PA diary over a seven day period. Participants cited ‘lack of enthusiasm’ and becoming ‘bored’ with the research as reasons why they did not wear the GPS and heart rate device for the full seven day period. It is widely recognised that the use of GPS (combined with other methods) also presents a high researcher burden [27] and the process of managing, cleaning and interpreting the wealth of data that this method provides is still being explored and developed. There was no participant reactivity found for the first day of testing, and based on the current study findings and experience, the traditional four day testing period as suggested by Trost *et al*. [63] is recommended for future research utilising GPS when measuring young people’s PA patterns. 

Given the explorative nature of the study in investigating the feasibility of utilising GPS to measure adolescents’ free-living PA over a full 7 day period, a relatively small sample was employed due to the logistical challenges presented by such a study. Hence, this acknowledged limitation of sample size in the current study should be carefully considered in future research. Employing a larger and more representative sample size should help in supporting the tentative conclusions drawn from this study. Whilst monitoring the PA patterns of youth within their surrounding BE using GPS provides challenges to researchers aiming to conduct large scale epidemiological studies, it is envisaged and hoped that with advancing technology and growing researcher expertise, such large scale projects utilising GPS will be achievable. The current study was limited to measuring young people’s PA levels during their free-time and subsequently does not explore the potentially significant opportunities for PA during the time youth spend in the school environment (such as during Physical Education lessons and recess). Finally, the cross-sectional design of the current study does not allow for exploring and establishing causal effects. Future longitudinal research incorporating GPS to measure geographical differences in young people’s PA would be highly insightful. Despite such limitations, however, the study achieved its primary aim of exploring the depth of information that GPS can provide in tracking young people’s PA patterns over a seven day period, outlining several potential PA pattern differences in young people from different geographical settings. 

## 5. Conclusions

The utilisation of GPS combined with heart rate monitoring and an activity diary provided a wealth of insightful information regarding the PA patterns of suburban and rural youth. The data collected indicated that suburban youth participated in more PA over the 7 day testing period (especially during weekdays). They also visited more public recreational facilities (such as parks and playgrounds), spent more time outdoors and participated in more PA when outdoors in their local neighbourhood environment. In comparison to the rural environment, the suburban environment provided youth with a broader range of recreational facilities which promoted PA, such as public parks, playing fields and playgrounds. In light of these findings, it can be implied that the suburban environment is more supportive for PA in youth. Throughout the results, rural females were substantially and consistently the least active subgroup and it may be beneficial for future research and policies to target this subgroup of youth when aiming to promote PA for health. This study is explorative in outlining tentative findings that may have implications and relevance on a larger more representative scale. The potential in utilising GPS to explore how distinct BE characteristics influence the behavioural and PA patterns of youth from differing geographic and demographic backgrounds is clearly apparent.
